# The Effect of Multilayer Nanoemulsion on the In Vitro Digestion and Antioxidant Activity of β-Carotene

**DOI:** 10.3390/antiox13101218

**Published:** 2024-10-10

**Authors:** Mei Zi Sun, Do-Yeong Kim, Youjin Baek, Hyeon Gyu Lee

**Affiliations:** 1 Department of Food and Nutrition, Hanyang University, 222, Wangsimni-ro, Seongdong-gu, Seoul 04763, Republic of Korea; sunmeizi27@daum.net (M.Z.S.); jyyj161126@hanyang.ac.kr (Y.B.); 2 Division of Food and Nutrition, Chonnam National University, Gwangju 61186, Republic of Korea; kimdy@jnu.ac.kr

**Keywords:** β-carotene, multilayer nanoemulsions, in vitro digestion, antioxidant

## Abstract

The objectives of this study were to design multilayer oil-in-water nanoemulsions using a layer-by-layer technique to enhance the stability of β-carotene and evaluate its effect on in vitro release and antioxidant activity. To prepare β-carotene-loaded multilayer nanoemulsions (NEs), a primary NE (PRI-NE) using Tween 20 was coated with chitosan (CS) for the secondary NE (SEC-CS), and with dextran sulfate (DS) and sodium alginate (SA) for the two types of tertiary NEs (TER-DS, TER-SA). The multilayer NEs ranged in particle size from 92 to 110 nm and exhibited high entrapment efficiency (92–99%). After incubation in a simulated gastrointestinal tract model, the release rate of free fatty acids decreased slightly after coating with CS, DS, and SA. The bioaccessibility of β-carotene was 7.02% for the PRI-NE, 7.96% for the SEC-CS, 10.88% for the TER-DS, and 10.25% for the TER-SA. The 2,2-diphenyl-1-picrylhydrazyl radical scavenging abilities increased by 1.2 times for the multilayer NEs compared to the PRI-NE. In addition, the cellular antioxidant abilities improved by 1.8 times for the TER-DS (87.24%) compared to the PRI-NE (48.36%). Therefore, multilayer nanoemulsions are potentially valuable techniques to improve the stability, in vitro digestion, and antioxidant activity of β-carotene.

## 1. Introduction

Carotenoids are natural pigments found at appreciable levels in many colored fruits and vegetables, including carrots, pumpkins, tomatoes, mangoes, kale, and broccoli [1]. β-Carotene has attracted particular attention amongst the carotenoid family due to its abundance in nature and strong bioactivities. An appropriate intake of β-carotene is generally considered beneficial to human health due to its unique bioactive attributes as an antioxidant and as a precursor of vitamin A [2,3,4]. Several studies have suggested that β-carotene plays an important role in preventing chronic diseases, improving eye health, reducing the risk of diabetes, and strengthening the immune system [5]. However, β-carotene cannot be synthesized within the human body, and therefore, the only source of β-carotene is the diet.

Nonetheless, the nature of its chemical structure, with a long carbon chain, makes β-carotene a hydrophobic molecule that is practically water insoluble [2]. In addition, β-carotene is notoriously known to be chemically unstable due to its highly susceptible double bonds, and it degrades in response to environmental stressors, such as pH, ionic strength, and temperature, which lead to its poor bioavailability [6]. Therefore, it remains challenging to incorporate β-carotene into many foods.

It is highly desirable to develop a delivery system which can both effectively encapsulate and provide protection for β-carotene from external influences, to fully realize its potential benefits. To overcome its shortcomings in food application, some encapsulation methods have been studied, such as encapsulating β-carotene into oil-in-water emulsions, liposomes, micelles, and biocompatible polymer complexes. Among these delivery systems, the oil-in-water emulsion system is the most widely adopted owing to its simplicity and high encapsulation efficiency. The low solubility of β-carotene can be circumvented by using oil as the solvent. Apart from being a mere solvent, lipids also improve the bioaccessibility of β-carotene when they are co-ingested. Nanoemulsions are particularly effective in improving the water dispersibility and bioaccessibility of β-carotene; however, they are less effective in improving its chemical stability. Single-layer nanoemulsions are susceptible to environmental stressors (e.g., heat, light, pH, oxygen, and free radicals), thus limiting their use in the food and pharmaceutical industries [7]. Therefore, many researchers have developed more complex emulsion systems that are specifically designed to improve the stability of packaged components. The most economical and effective methods involve multilayer emulsions.

In the multilayer oil-in-water emulsion system, β-carotene is insulated from the external environment by the emulsifier layer so that it can be more stable against external stimuli. More importantly, the triggered release of β-carotene is made possible by manipulating the properties of the polyelectrolyte used to construct the interface, which makes oil-in-water emulsion an ideal candidate for the delivery of β-carotene in complex food systems. Functional ingredients, such as antioxidants and antimicrobials, can be incorporated into multicoat materials.

Typically, β-carotene-loaded oil-in-water emulsions are created when β-carotene is first dissolved within an oil phase, which is then brought under homogenization together with an aqueous phase in the presence of a surfactant at a suitable proportion [8]. This generates dispersed oil droplets containing β-carotene and an interface accumulated with surfactant molecules to stabilize the emulsion droplets and provide insulation between the oil and aqueous phases. However, previous studies have found that emulsions stabilized using a single-component emulsifier are so unstable that they fail to cater to the requirement for β-carotene delivery due to the complex chemical conditions within the human body [9]. The results of previous studies indicate that β-carotene encapsulated in small emulsifiers, such as Tween 20 or fatty acids, undergoes considerable degradation over time in spite of successful encapsulation. Protein-stabilized emulsions, on the other hand, are vulnerable to destabilization near the isoelectric point of the proteins, at which point they become charge-neutral and precipitate out [5,10,11].

In addition to the normal oil-in-water emulsion method, attempts have been made to assemble multiple functional layers on oil droplets. This is typically achieved by depositing biopolymer layers through electrostatic interactions between the different layers. Standard layer-by-layer electrostatic deposition normally involves two stages: the formation of primary emulsions and the subsequent absorption of the secondary layer carrying an opposite charge, after which the net charge of the bilayer droplets is reversed. The absorption procedure is repeated by depositing another oppositely charged polyelectrolyte layer onto the bilayer droplets. Emulsions stabilized using multiple functional layers are more stable than normal emulsions against external stimuli, such as ionic strength, pH, and temperature fluctuations [12]. Previous studies have shown that the positively charged biopolymeric surfactant chitosan (CS) has the dual functionality of providing additional stability to emulsions stabilized by small-molecule surfactants, such as sodium dodecyl sulfate and lecithin [9,13,14]. However, the deposition of an additional polyelectrolyte layer is not always favorable because the interaction between different biopolymer layers is mainly determined by electrostatic attraction and steric repulsion. When biopolymers do not possess sufficient charge, steric repulsion prevails, preventing the formation of a multilayer emulsion. Therefore, it is necessary to investigate the types and concentrations of different biopolymers when preparing multilayer emulsions. However, there are few studies aimed at developing β-carotene-loaded oil-in-water multilayer nanoemulsions. In this study, we used dextran sulfate (DS) and sodium alginate (SA) as a negatively charged biopolymer as coating materials. DS has sulfate residue which can enhance its mucoadhesive property via strong hydrogen interaction with the mucus layer [15]. Moreover, SA is also known for its mucoadhesive property through hydrogen interaction [16].

Therefore, the aim of this study was to design a β-carotene-loaded oil-in-water multilayer nanoemulsion-based delivery system. For this purpose, the relationship between different multilayer coatings suitable for food use and their concentrations was studied to improve the stability of both emulsions and β-carotene. CS, dextran sulfate sodium salt (DS), and alginic acid sodium salt (SA) were used as functional multilayer coatings to prepare the β-carotene-loaded multilayer nanoemulsions. The effects of multilayer nanoemulsions on antioxidant ability, release characteristics, lipid digestion, and in vitro bioaccessibility were also evaluated.

## 2. Materials and Methods

### 2.1. Materials

Corn oil was purchased from a supermarket (Seoul, South Korea). β-Carotene, Tween 20, DS, SA, porcine bile extract, calcium chloride, lipase from porcine pancreas, 3,4,5-dimethylthiazol-2-yl-2-5-diphenyltetrazolium bromide (MTT), 2,2-diphenyl-1-picrylhydrazyl (DPPH), and 2,2′-azobis(2-methylpropionamidine) dihydrochloride (AAPH) were purchased from Sigma-Aldrich Chemical Co. (St Louis, MO, USA). CS (water soluble, 24 cps, 95% deacetylated) was obtained from Kittolife Co. (Seoul, South Korea). All the other chemicals were of analytical grade.

The colon carcinoma cell line (Caco-2) and HEK293 cells (human embryonic kidney cells) were obtained from the Korean Cell Line Bank (Seoul, South Korea). Minimum essential medium (MEM), fetal bovine serum (FBS), MEM non-essential amino acids (MEM-NEAA), penicillin-streptomycin, Hanks’ balanced salt solution (HBSS), and 0.25% trypsin-EDTA were purchased from Gibco Life Technologies (Mulgrave, Australia).

### 2.2. Preparation of β-Carotene-Loaded Nanoemulsions

#### 2.2.1. Preparation of the Primary Nanoemulsion

An oil phase was prepared by dispersing 0.1% (*w*/*w*) β-carotene into corn oil, heating at 50 °C (<5 min), and stirring. A coarse emulsion was prepared by homogenizing a 4% (*w*/*w*) oil phase with a 96% (*w*/*w*) aqueous emulsifier solution (1.5% [*w*/*w*] Tween 20, phosphate buffer) using a high-speed homogenizer (HG-15A; Daihan Scientific Co., Ltd., Seoul, South Korea) at 10,800 rpm for 3 min. The coarse emulsion was passed through a microfluidizer three times (LM 20; Microfluidics, Newton, MA, USA), operating at 10,000 psi.

#### 2.2.2. Preparation of the Secondary Nanoemulsion

CS solutions were prepared using deionized water at concentrations of 0.0078–1.0% (*w*/*w*). To prepare the SEC-CS, the PRI-NE was added dropwise to different concentrations of CS solutions (0.0078–1.0000% (*w*/*w*)) at a 1:1 volume ratio, with stirring at 1000 rpm for 10 min.

#### 2.2.3. Preparation of Tertiary Nanoemulsions

DS and SA solutions were prepared by dispersing 0.05–0.15% (*w*/*w*) DS and SA in deionized water. To prepare the TER-DS and TER-SA, the SEC-CS was added dropwise to different concentrations of DS and SA solutions (0.05–0.15% (*w*/*w*)) at a 1:1 volume ratio, with stirring at 1000 rpm for 10 min.

### 2.3. Physical Properties of the Nanoemulsions

In order to analyze the formation of the PRI-NE, SEC-CS, TER-DS, and TER-SA, the particle size, polydispersity index (PDI), derived count rate (DCR), and zeta potential of β-carotene-loaded nanoemulsions were determined using a Zetasizer Nano ZS (Malvern Instruments Ltd., Malvern, UK). Specifically, the successful formation of secondary and tertiary coating layers was confirmed through the changes in zeta potential values. Each nanoemulsion sample was measured in multiple narrow modes at 25 ± 1 °C. The samples were diluted 100-fold with deionized water.

### 2.4. Determination of Entrapment Efficiency

The entrapment efficiency (EE) of β-carotene-loaded nanoemulsions was determined using the Amicon Ultra-15 (Millipore Co., Burlington, MA, USA). After centrifugation of the β-carotene-loaded nanoemulsion at 4000× *g* for 15 min at 25 °C, the amount of free β-carotene in the filtered solutions was analyzed. The aliquots of filtered solutions were mixed with chloroform at a volume ratio of 1:1. The β-carotene extracts were then analyzed using a UV-Vis spectrophotometer (GEN 10S UV-Vis; Thermo Fisher scientific, Waltham, MA, USA) at 450 nm. EE was calculated using the following Equation (1) [17]:

EE (%) = (total amount − free amount in supernatant)/total amount × 100(1)

where the total amount is the total amount of β-carotene in nanoemulsions and the free amount in supernatant is the free amount of β-carotene in the supernatant.

### 2.5. Cytotoxicity

The cytotoxicity of the β-carotene nanoemulsions was determined using the MTT assay, which is based on the reduction of MTT into a purple formazan product by mitochondrial dehydrogenase in intact cells [18]. The HEK293 cells were seeded in 96-well plates at a density of 1 × 10^4^ cells/well. After 24 h, the cells were treated with 20 μL of the nanoemulsions and incubated for 24 h. After the treatment, 20 μL of MTT solution (5 mg/mL in PBS) was added to each well. The plates were incubated for 4 h and centrifuged at 217× *g* for 5 min. The medium was then removed and 150 μL of dimethyl sulfoxide was added to each well to dissolve the formazan crystals. The absorbance was measured at 540 nm using a Synergy HT multi-microplate reader (BioTek Instruments, Winooski, VT, USA).

### 2.6. In Vitro Lipid Digestion

The in vitro simulated gastrointestinal tract (GIT) model consisted of gastric and intestinal phases. Simulated gastric fluid (SGF) was prepared by dissolving 2 g of NaCl in 0.26% HCl. The pH was adjusted to 1.2 using 1.0 N HCl. The NE sample was then mixed with SGF at a 1:1 volume ratio, and the pH of the mixture was adjusted to 2.5 using 1 M NaOH. Then, it was incubated at 37 °C in a water bath for 2 h, with shaking at 100 rpm. Subsequently, 30 mL of the digested sample was adjusted to 7.0 and then mixed with an intestinal enzyme complex solution (4 mL of 5 mg/mL bile extract solution, 110 mg of CaCl_2_ dissolved in 1 mL distilled water, and 15 mg of lipase dissolved in 2.5 mL phosphate buffer) at a 1:1 volume ratio in a water bath at 37 °C, with shaking at 100 rpm [19].

After incubation, the physical properties of the digested samples were determined using a Zetasizer Nano ZS instrument (Malvern Instruments Ltd., Malvern, UK). Moreover, the amount of free fatty acids (FFAs) released from NE samples during lipid digestion was evaluated. Briefly, the amounts of 0.1 M NaOH solution added to the digested nanoemulsion solutions in SGF and SIF to maintain pH at 2.5 and 7.0, respectively, were analyzed. These amounts of 0.1 M NaOH solution were assumed to be related to the amount of FFAs released during lipid digestion. The amount of FFA released was calculated from the titration curves using Equation (2), as described previously [19]:

FFA (%) = (V_NaOH_ × C_NaOH_ × W_Lipid_)/(2 × w_Lipid_) × 100 (2)

where V_NaOH_ is the volume of NaOH solution required to neutralize the FFAs produced after digestion time (L), C_NaOH_ is the molarity of the NaOH solution used to titrate the sample (M), M_Lipid_ is the molecular weight of the oil (g·mol^−1^), and w_Lipid_ is the total mass of oil initially present in the incubation cell (g).

### 2.7. In Vitro Bioaccessibility of β-Carotene

The in vitro bioaccessibility of β-carotene was determined using a previously described method [20]. Briefly, after undergoing a lipid digestion process as described in Section 2.6, an aliquot of lipid-digested solution was centrifuged at 3470× *g* for 40 min at 25 °C. This resulted in separation into an oily phase at the top, a clear micelle phase in the middle, and an opaque sediment phase at the bottom. Then, 5 mL of the clear micelle phase and lipid-digested solution without centrifugation were mixed with chloroform at equal volume and centrifuged at 664× *g* for 10 min. The bottom layer containing solubilized β-carotene was collected, and the aliquots of the above solution were analyzed using a UV-Vis spectrophotometer at 450 nm to evaluate the concentration of β-carotene in the clear micelle phase and lipid-digested solution. The in vitro bioaccessibility of β-carotene was calculated using the following Equation (3) [20]:

Bioaccessibility (%) = C_Micelle_/C_RawDigesta_ × 100 (3)

where C_Micelle_ is the concentration of β-carotene in the micelle fraction after lipolysis and C_RawDigesta_ is the concentration of β-carotene in the lipid-digested solution after lipolysis.

### 2.8. Antioxidant Activity

#### 2.8.1. DPPH Radical Scavenging Assay

A 50 μL aliquot of the nanoemulsion was mixed with 150 μL of 0.36 mM DPPH and incubated at 25 °C. After 45 min of reaction, the absorbance was measured at 517 nm using a Synergy HT multi-microplate reader. DPPH radical scavenging activity was calculated using the following Equation (4):

DPPH radical scavenging effect (%) = (C − [S − SB]) × 100(4)

where C is the absorbance of the control (mixture of DPPH solution and ethanol), S is the absorbance of the sample, and SB is the absorbance of the blank.

#### 2.8.2. Cellular Antioxidant Activity Assay

Caco-2 cell suspensions were seeded at a density of 6 × 10^4^ cells/well in black 96-well microplates. The growth medium was removed after 24 h of incubation, and the cells were washed once with 100 μL of PBS. Then, 50 μL of each sample and 50 μL of 25 μM dichloro-dihydro fluorescein diacetate (DCFH-DA), which was dissolved in growth medium, was added to the cells. After 1 h of reaction, the reaction solution was removed, and the cells were washed with 100 μL of PBS. An aliquot of 100 μL of 600 μM 2,2′-azobis[2-amidinopropane] dihydrochloride (ABAP) dissolved in HBSS was then applied to the cells. The microplate was placed into a Synergy HT multi-microplate reader to measure the fluorescence (emission, 538 nm; excitation, 485 nm) for 60 min at 37 °C. The cellular antioxidant activity (CAA) was calculated using the following Equation (5) [21]:

CAA unit = 1 − ∫SA/∫CA(5)

where ∫SA is the integrated area under the sample fluorescence versus time curve and ∫CA is the integrated area under the control curve.

### 2.9. Statistical Analysis

All experiments were performed in triplicate, and the results are expressed as the mean and standard deviation. Statistical Package for the Social Sciences (SPSS, Version21.0, SPSS Inc., Chicago, IL, USA) was used to perform the analyses. Statistical analysis was performed using one-way analysis of variance (ANOVA) followed by Duncan’s multiple range test, and a significant difference was determined as *p* < 0.05.

## 3. Results and Discussion

### 3.1. Physical Properties of the Nanoemulsions

#### 3.1.1. Characterization of PRI-NE

The coarse emulsion was prepared by homogenizing the oil phase (β-carotene and corn oil) and the aqueous phase (phosphate buffer and Tween 20). It was then passed through a high-pressure microfluidizer to obtain the PRI-NE. The average particle size was approximately 92.2 nm. This was similar to previously reported results [19,22]. The PDI value indicates the distribution of particle sizes [20]. A PDI value less than 0.3 indicates a monodispersed system, while a PDI value greater than 0.7 indicates a polydispersed system [23]. The average PDI value of the PRI-NE was 0.163 ± 0.013, indicating that the particles had a uniform size distribution. The zeta potential indicates the degree of electrostatic repulsion between adjacent similarly charged particles in colloidal dispersions [24]. A zeta potential with an absolute value greater than 30 mV indicates a stable suspension of particles [25]. The average zeta potential of the PRI-NE was −42.2 ± 1.7, indicating that it had good stability. The DCR represents the scattered light intensity of the particles and can also be used to determine the particles’ relative concentration [26]. In other words, increased DCR demonstrates an increase in the number of the particles. In this study, the DCR of the PRI-NE was of a high value, indicating that the PRI-NE was formulated with a high amount of particles of small particle size [27].

#### 3.1.2. Influence of CS Concentration on the Characteristics of SEC-CS

To investigate the effect of CS concentration on the physical properties of SEC-CSs, SEC-CSs were prepared by dropping PRI-NE into different concentrations of CS solutions in the range of 0.0078–1.0% (*w*/*w*). Physical properties such as particle size, PDI value, zeta potential, and DCR of the SEC-CSs were determined.

**Figure 1:**
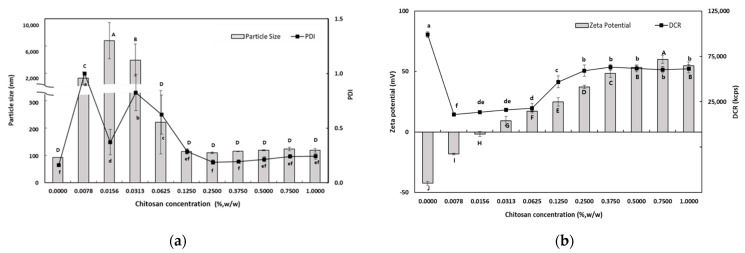
Dependence of the particle size (**a**), PDI (**a**), DCR (**b**), and zeta potential (**b**) on chitosan concentration for primary nanoemulsions. Significant differences in the primary nanoemulsion’s particle size or zeta potential values depending on the concentration of CS were demonstrated with different capital letters (*p* < 0.05). Significant differences in the primary nanoemulsion’s PDI or DCR depending on the concentration of CS were demonstrated with different lowercase letters (*p* < 0.05). Measurement was performed in triplicate, and the results are expressed as the mean and standard deviation.

Figure 1a shows the dependence of the particle size on the CS concentration of the PRI-NE. At lower CS concentrations (0.0078–0.0313%, *w*/*w*), there was a significant increase in the mean particle size. The mean particle size was approximately a few microns at CS concentrations of 0.0078–0.0313% (*w*/*w*), and the PDI values were greater than 0.3. This may be interpreted as an effect of the interaction of CS with PRI-NE on the surface of the particles at low concentrations, resulting in destabilization due to charge neutralization. This charge neutralization induces the particles to bind more tightly, promoting the formation of larger aggregates. This is also because CS is not sufficient to cover all of the PRI-NE particles. The increased particle size may be due to bridging flocculation of the PRI-NE droplets induced by CS. This phenomenon was due to the aggregation of oil droplets, resulting in an increase in both the particle size and the PDI value. This is similar to the results of a previous study [28]. However, at higher concentrations of CS (0.0625–1.0%, *w*/*w*), there was a relative decrease in the mean particle size of the SEC-CSs. This can be explained by the strong electrostatic repulsion and steric hindrance between the SEC-CS droplets, thus inhibiting flocculation. This was sufficient to cover the droplets and form an adequately thick interfacial layer on the PRI-NE droplets as the CS concentration increased. Therefore, both the particle size and the PDI value of the SEC-CSs decreased [29]. Previous studies by many researchers have reported similar results [28,30,31].

When 0.25% (*w*/*w*) CS was added, the particle size was approximately 110 nm and the PDI value was 0.189. The difference in particle size between the SEC-CS (0.25% *w*/*w*) and the PRI-NE indicated that CS changed the adsorption properties of the PRI-NE [32].

The influence of CS on the zeta potential of the SEC-CSs is shown in Figure 1b. The zeta potential of the PRI-NE was −42.4 ± 1.7 mV, and as the CS concentration increased, the zeta potential of the SEC-CSs became increasingly positive. This indicates that by forming a Tween 20–CS film, positively charged CS molecules were adsorbed onto the surface of the negatively charged PRI-NE droplets. When the CS concentration exceeded 0.25% (*w*/*w*), the zeta potential reached a constant value of more than +30 mV. This suggests that the PRI-NE droplets were saturated with CS. Not only did the zeta potential reach a stable value above 30 mV, but the DCR was of a consistent value. The optimum CS concentration for SEC-CS was selected to be 0.25% (*w*/*w*) as the smallest particle size (109.5 nm) with a PDI value of 0.189 and a zeta potential of +37.3 mV was obtained at this concentration, indicating a stable system. Therefore, we produced SEC-CS with a CS concentration of 0.25% (*w*/*w*) for preparation of the TER-DS and TER-SA, and for further analysis.

#### 3.1.3. Influence of DS and SA Concentrations on the Characteristics of TER-DS and TER-SA

The purpose of these experiments was to identify the optimum concentration for preparing TER-NEs via layer-by-layer electrostatic deposition. The TER-NEs (TER-DS and TER-SA) were prepared by dropping SEC-CS into DS and SA solutions. To select the optimum DS and SA concentrations for TER-NE preparation, various TER-NEs were prepared with different DS and SA concentrations. Ideally, CS-coated lipid droplets should be covered with DS or SA without promoting bridging or depletion flocculation. Physical properties such as particle size, PDI value, zeta potential, and DCR of the TER-NEs were determined.

**Figure 2:**
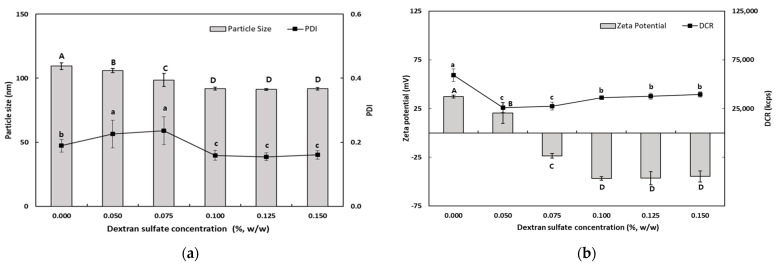
Dependence of the particle size (**a**), PDI (**a**), DCR (**b**), and zeta potential (**b**) on dextran sulfate concentration for secondary nanoemulsions at a chitosan concentration of 0.25% (*w*/*w*). Significant differences in the primary nanoemulsion’s particle size or zeta potential values depending on the concentration of CS were demonstrated with different capital letters (*p* < 0.05). Significant differences in the primary nanoemulsion’s PDI or DCR depending on the concentration of CS were demonstrated with different lowercase letters (*p* < 0.05). Measurement was performed in triplicate, and the results are expressed as the mean and standard deviation.

As shown in Figure 2, the particle size of TER-DSs coated with DS solution (0.05–0.15%, *w*/*w*) decreased when the concentration of DS increased. The mean particle size of the TER-DSs decreased to 91.9 nm as the DS concentration increased from 0.5 to 1.0% (*w*/*w*). The PDI values of the TER-DSs decreased as the DS concentration increased. The zeta potential became increasingly negative as DS was added to the emulsions. When the DS concentration reached 1.0% (*w*/*w*), the zeta potential became a constant value of −46.6 ± 1.9 mV, indicating that the SEC-CS droplets became fully saturated with DS.

**Figure 3:**
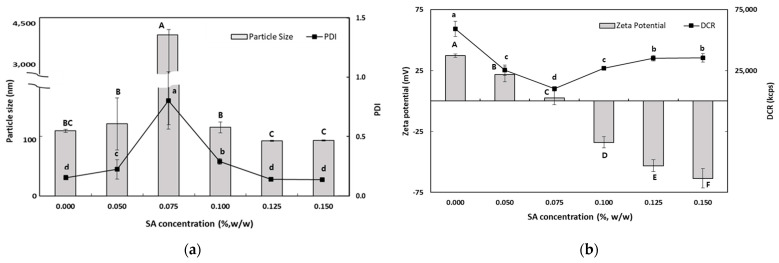
Dependence of the particle size (**a**), PDI (**a**), DCR (**b**), and zeta potential (**b**) on SA concentration for secondary nanoemulsions at a chitosan concentration of 0.25% (*w*/*w*). Significant differences in the primary nanoemulsion’s particle size or zeta potential values depending on the concentration of CS were demonstrated with different capital letters (*p* < 0.05). Significant differences in the primary nanoemulsion’s PDI or DCR depending on the concentration of CS were demonstrated with different lowercase letters (*p* < 0.05). Measurement was performed in triplicate, and the results are expressed as the mean and standard deviation.

As the SA concentration increased, the electrical charge on the TER-SA droplets became increasingly negative (Figure 3), suggesting that negatively charged SA molecules were adsorbed onto the surface of the positively charged SEC-CS droplets, leading to the formation of CS–SA interfacial layers.

When the SA concentration exceeded 0.1% (*w*/*w*), the absolute zeta potential exceeded 30 mV, indicating that the TER-NE droplets were fully saturated with SA. The particle size (92.6 ± 1.0 nm) and the PDI value (0.176 ± 0.009) of TER-SA were determined to be optimal at an SA concentration of 0.125% (*w*/*w*). At SA concentrations below 0.1% (*w*/*w*), an increase in particle size was observed. This is known as a bridge-aggregation phenomenon, in which two or more SEC-CS droplets are linked by SA bridges, resulting in the formation of rapidly creamed large aggregates [33].

#### 3.1.4. Characterization of the Nanoemulsions

Table 1 shows the physical properties of the PRI-NE, SEC-CS, TER-DS, and TER-SA prepared under the optimum conditions, and their particle sizes, PDI values, zeta potentials, and DCR were compared. With the addition of CS, the particle size of the SEC-CS increased from 92 to 110 nm and the zeta potential changed from highly negative (−42 mV) to positive (+37 mV), indicating that CS formed a coating layer on the surface of the PRI-NE. These results are similar to those of previous studies [34,35]. The PDI values of the PRI-NE and SEC-CS were 0.163 and 0.189, respectively. The low PDI values indicated that PRI-NE and SEC-CS particle sizes were distributed in a narrow range. As the DS or SA solution was added to the SEC-CS, the particle size of the TER-DS and TER-SA decreased to 91.9 nm and 92.6 nm, respectively; the zeta potential changed from positive to highly negative; and the PDI value remained <0.200. This can be interpreted as particle shrinkage caused by the strong ionic attraction between CS and DS/SA, forming a thin double layer [15].

### 3.2. Entrapment Efficiency

**Figure 4:**
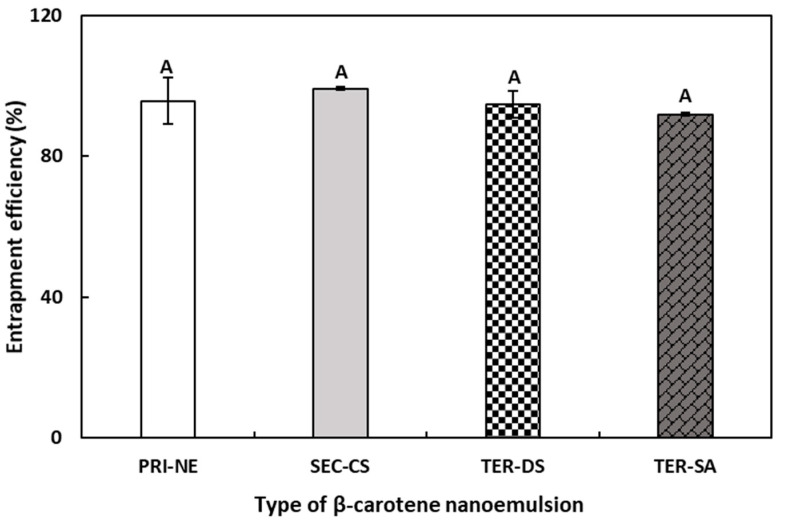
EE of PRI-NE, SEC-NE, TER-DS, and TER-SA. ^A^ Mean with letter is significantly different (*p* < 0.05). Key: PRI-NE = primary nanoemulsion; SEC-CS = secondary nanoemulsion coated with chitosan; TER-DS = tertiary nanoemulsion coated with dextran sulfate sodium salt; TER-SA = tertiary nanoemulsion coated with alginic acid sodium salt. Measurement was performed in triplicate, and the results are expressed as the mean and standard deviation.

The EE results were indirectly calculated by quantifying the amount of unencapsulated free β-carotene in the NEs. Originally, the total amounts of β-carotene in the PRI-NE, SEC-CS, and TER nanoemulsion were 0.04, 0.02, and 0.01 mg/g, respectively. As shown in Figure 4, the EE values of the PRI-NE, SEC-CS, TER-DS, and TER-SA were greater than 90%. The EE values of the TER-DS and TER-SA did not demonstrate any significant differences compared to those of the PRI-NE and SEC-CS. This may be explained by the finding that shrinkage caused by high electrostatic attraction between multiple coatings did not influence the EE of the multilayer nanoemulsions [31].

### 3.3. Cytotoxicity of the Nanoemulsions

**Figure 5:**
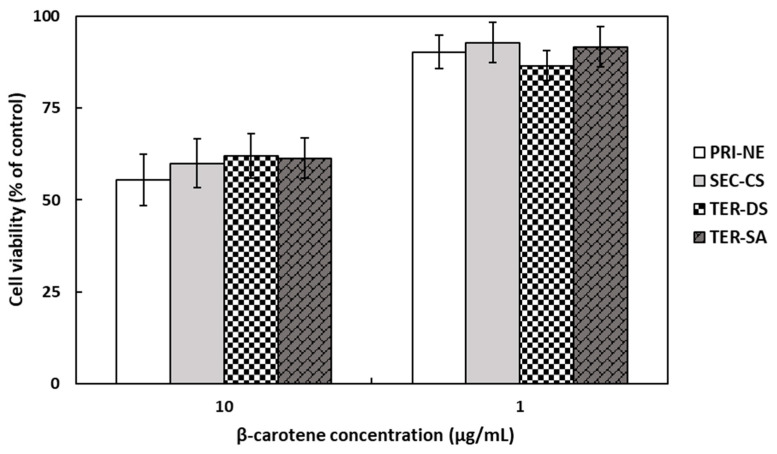
Cell viabilities of HEK293 cells treated with β-carotene-loaded multilayer nanoemulsions. Key: PRI-NE = primary nanoemulsion; SEC-CS = secondary nanoemulsion coated with chitosan; TER-DS = tertiary nanoemulsion coated with dextran sulfate sodium salt; TER-SA = tertiary nanoemulsion coated with alginic acid sodium salt. Measurement was performed in triplicate, and the results are expressed as the mean and standard deviation.

The in vitro cytotoxicity of β-carotene-loaded nanoemulsions on HEK293 cell monolayers was evaluated using the MTT assay (Figure 5), which is a colorimetric method that measures metabolic activity in living cells based on the reduction of the tetrazolium dye in MTT into purple crystals [36]. The cells were treated with different types of nanoemulsions at concentrations of 1 and 10 μg/mL. At a β-carotene concentration of 10 μg/mL, the average cell viability was approximately 60% of the viability of non-treated control cells. At a lower β-carotene concentration of 1 μg/mL, the number of viable cells present was more than 86%. However, it is worth noting that cell viability was not influenced by the type of nanoemulsion.

### 3.4. In Vitro Digestion

#### 3.4.1. Influence of Initial Nanoemulsion Type on the Size and Electrical Characteristics

**Figure 6:**
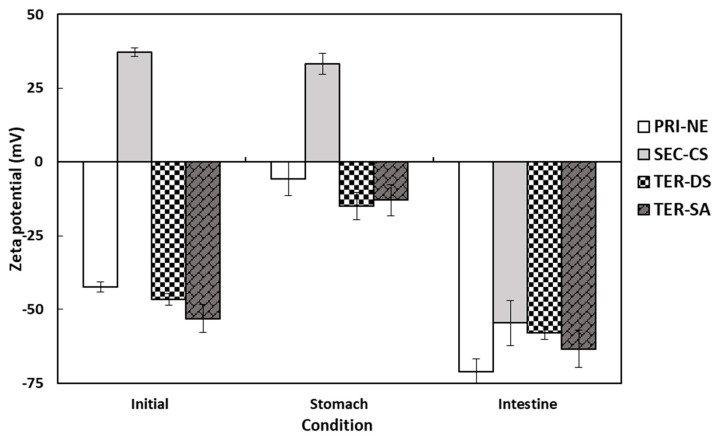
Influence of simulated gastrointestinal conditions on the zeta potential of β-carotene-loaded multilayer nanoemulsions. Key: PRI-NE = primary nanoemulsion; SEC-CS = secondary nanoemulsion coated with chitosan; TER-DS = tertiary nanoemulsion coated with dextran sulfate sodium salt; TER-SA = tertiary nanoemulsion coated with alginic acid sodium salt. Measurement was performed in triplicate, and the results are expressed as the mean and standard deviation.

The electrical characteristics of the PRI-NE, SEC-CS, TER-DS, and TER-SA particles followed similar trends after passing through various regions of the in vitro GIT model (Figure 6). The initial PRI-NE had a negative surface charge (−42 mV). Some studies have shown that fat droplets stabilized by non-ionic surfactants may have a significant negative charge, owing to the preferential adsorption of hydroxyl ions from the aqueous phase or the presence of anionic impurities (e.g., FFAs) in the surfactant or oil used to prepare the emulsion [37]. The zeta potentials of the SEC-CS, TER-DS, and TER-SA samples were +37, −47, and −53 mV, respectively. The positive and negative charge changes were due to complete encapsulation of the coating materials. After exposure to the stomach phase, the absolute value of the zeta potential of all droplets decreased slightly. In turn, the zeta potential of all nanoemulsion droplets became significantly more negative after the small intestine phase. The values ranged from −55 to −71 mV.

The high negative charge may be due to the presence of surface-active anionic species in the SIF. These include bile salts, phospholipids, lipases, and FFAs [35,36,37,38]. Many researchers have reported similar trends in the electrical properties of oil-in-water emulsions after digestion [19,20,39,40,41,42].

**Figure 7:**
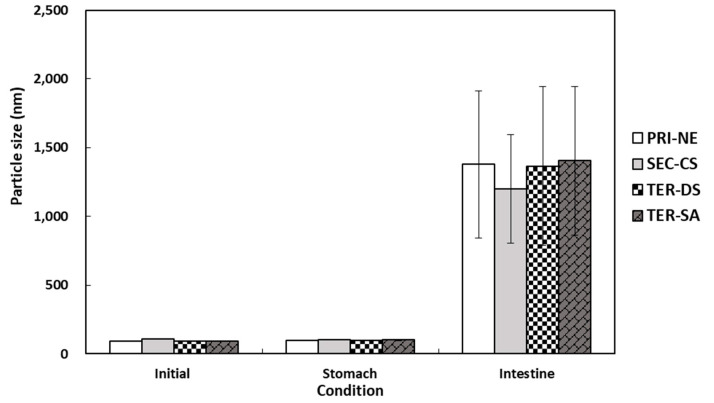
Influence of simulated gastrointestinal conditions on the mean particle size of β-carotene-loaded multilayer nanoemulsions. Key: PRI-NE = primary nanoemulsion; SEC-CS = secondary nanoemulsion coated with chitosan; TER-DS = tertiary nanoemulsion coated with dextran sulfate sodium salt; TER-SA = tertiary nanoemulsion coated with alginic acid sodium salt. Measurement was performed in triplicate, and the results are expressed as the mean and standard deviation.

The particle size distributions of the lipid droplets in the initial PRI-NE, SEC-CS, TER-DS, and TER-SA exhibited single peaks with particle sizes of 92, 110, 92, and 93 nm, respectively (Figure 7). After incubation for 2 h in SGF, the particle sizes of all nanoemulsions did not increase significantly, suggesting that they were relatively resistant to aggregation. This is consistent with the results of a previous study [19]. After incubation in SIF, the four types of nanoemulsions exhibited a large increase in the mean particle size, indicating that they were unstable in the face of droplet aggregation [43]. These nanoemulsions were largely released.

#### 3.4.2. Influence of Initial Nanoemulsion Type on Lipid Digestion

**Figure 8:**
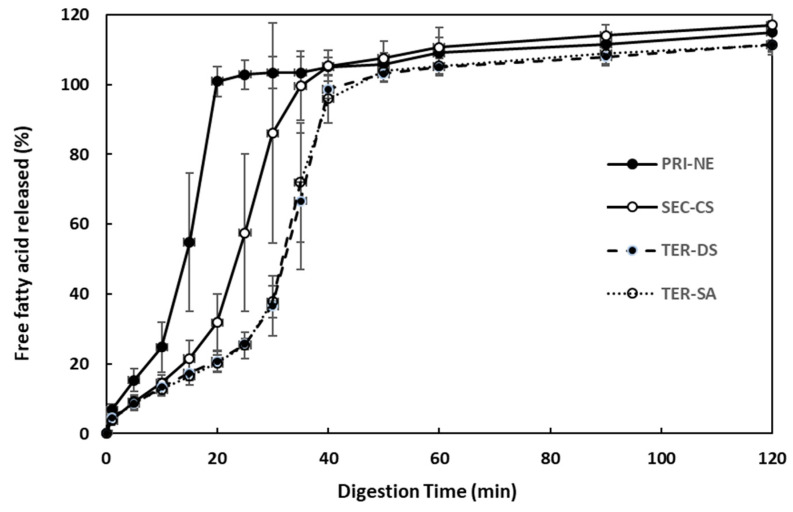
Influence of the types of multilayer nanoemulsions on in vitro digestion under simulated small intestinal conditions. Key: PRI-NE = primary nanoemulsion; SEC-CS = secondary nanoemulsion coated with chitosan; TER-DS = tertiary nanoemulsion coated with dextran sulfate sodium salt; TER-SA = tertiary nanoemulsion coated with alginic acid sodium salt. Measurement was performed in triplicate, and the results are expressed as the mean and standard deviation.

The effect of the initial nanoemulsion type on the rate and extent of triglyceride digestion was examined using the pH-stat method (Figure 8). The amount of FFA released increased steeply over 40 min under simulated intestinal conditions. Among all types of nanoemulsions, the PRI-NE showed the fastest (15 min) FFA release. The SEC-CS, TER-DS, and TER-SA released FFA at similar rates. These results indicated that the release rate was slightly controlled, because CS, DS, and SA formed layers on the surface of the oil droplets to protect them from degradation by digestive enzymes.

#### 3.4.3. Influence of Initial Nanoemulsion Types on β-Carotene Bioaccessibility

Typically, β-carotene has low bioavailability due to its water insolubility and chemical instability. To evaluate the effect of multilayer emulsions on bioavailability, the influence of the four types of nanoemulsions on the in vitro bioaccessibility of β-carotene was determined.

**Figure 9:**
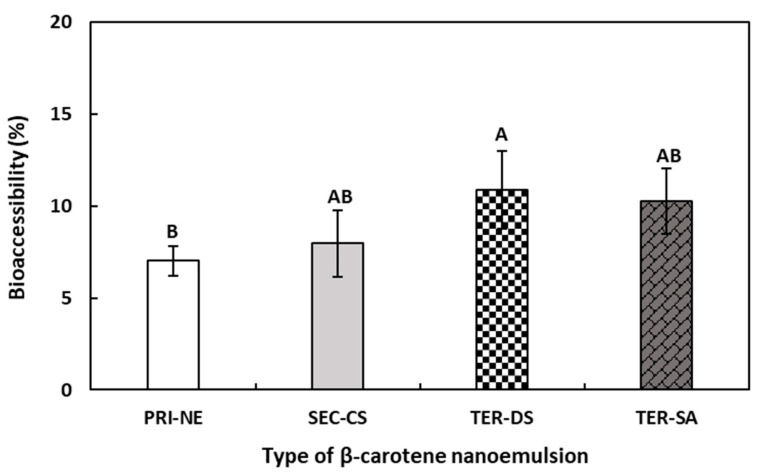
Influence of the types of multilayer nanoemulsions on the bioaccessibility (%) of β-carotene after in vitro digestion. ^A,B^ Means with different letters are significantly different (*p* < 0.05). Key: PRI-NE = primary nanoemulsion; SEC-CS = secondary nanoemulsion coated with chitosan; TER-DS = tertiary nanoemulsion coated with dextran sulfate sodium salt; TER-SA = tertiary nanoemulsion coated with alginic acid sodium salt. Measurement was performed in triplicate, and the results are expressed as the mean and standard deviation.

According to the results shown in Figure 9, the bioaccessibility of β-carotene decreased in the following order: TER-DS > TER-SA ≥ SEC-CS > PRI-NE. Although the difference in bioaccessibility between the nanoemulsions was relatively small, a difference was observed between the multilayer emulsions and the PRI-NE. The bioaccessibility of β-carotene in the PRI-NE, SEC-CS, TER-SA, and TER-DS was 7.02%, 7.96%, 10.25%, and 10.88%, respectively. The bioaccessibilities of the multilayer emulsions were 1.13-fold, 1.46-fold, and 1.55-fold higher than that of the PRI-NE. This could be attributed to the fact that the SEC-CS, TER-DS, and TER-SA were more stable in SGF because the oil drops were coated with layers of CS, CS-DS, and CS-SA. The SEC-CS, TER-DS, and TER-SA formed more stable structures, in which the lipase readily formed micelles in SIF, leading to higher in vitro bioaccessibility. Previous studies have reported that nanoemulsions increase the bioaccessibility of lipophilic bioactive agents [44,45,46]. However, there are few studies assessing differences in bioaccessibility between multilayer and normal nanoemulsions. Therefore, the effects of multilayer nanoemulsions and normal nanoemulsions on bioaccessibility require further study.

### 3.5. Antioxidant Activity

#### 3.5.1. DPPH Radical Scavenging Activity

**Figure 10:**
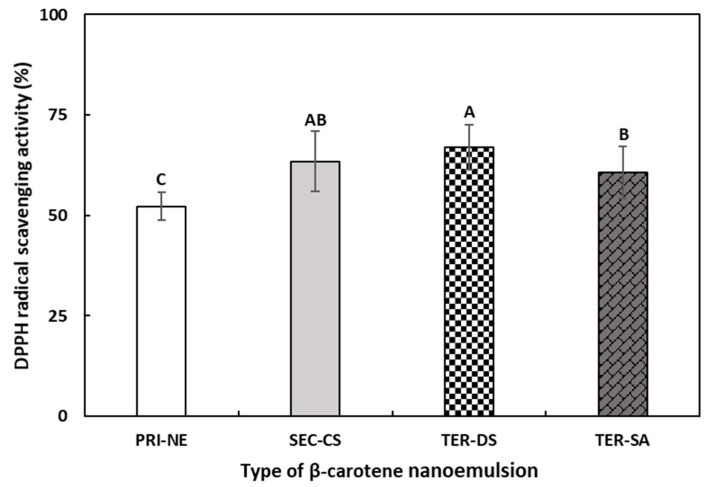
The DPPH radical scavenging activities of β-carotene-loaded multilayer nanoemulsions. ^A–C^ Means with different letters are significantly different (*p* < 0.05). Key: PRI-NE = primary nanoemulsion; SEC-CS = secondary nanoemulsion coated with chitosan; TER-DS = tertiary nanoemulsion coated with dextran sulfate sodium salt; TER-SA = tertiary nanoemulsion coated with alginic acid sodium salt. Measurement was performed in triplicate, and the results are expressed as the mean and standard deviation.

To evaluate the effects of the multicoat materials on antioxidant activity, the DPPH radical scavenging abilities of the PRI-NE, SEC-CS, TER-DS, and TER-SA were measured (Figure 10). The DPPH assay is commonly used to evaluate the ability of compounds to act as hydrogen donors and to scavenge free radicals [47,48].

The DPPH scavenging ability of all multilayer nanoemulsions was approximately 1.2 times higher than that of the PRI-NE. This may be explained by the antioxidant activity of biopolymers used as coating materials in this study [49,50]. In addition, the coating materials better protected β-carotene from degradation by environmental stressors [51,52].

#### 3.5.2. Cellular Antioxidant Activity

**Figure 11:**
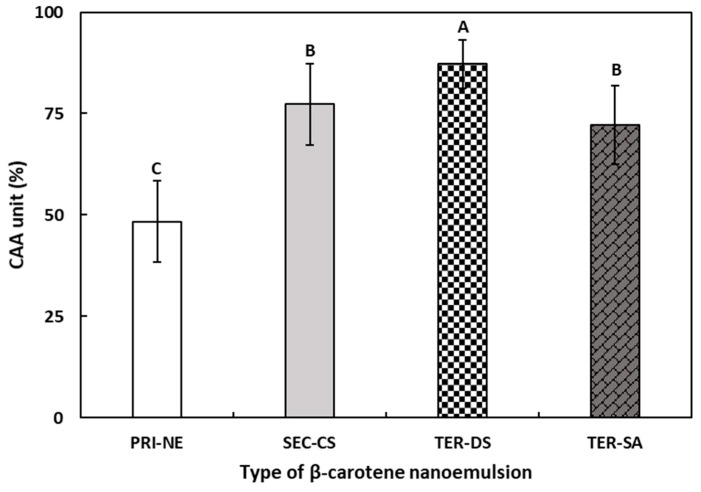
The CAA values of β-carotene-loaded multilayer nanoemulsions. ^A–C^ Means with different letters are significantly different (*p* < 0.05). Key: PRI-NE = primary nanoemulsion; SEC-CS = secondary nanoemulsion coated with chitosan; TER-DS = tertiary nanoemulsion coated with dextran sulfate sodium salt; TER-SA = tertiary nanoemulsion coated with alginic acid sodium salt. Measurement was performed in triplicate, and the results are expressed as the mean and standard deviation.

The CAA assay was performed to determine the antioxidant activity of the four types of β-carotene-loaded NEs (Figure 11). The CAA assay may be more representative of the complexity of biological systems than commonly used antioxidant activity assays [53].

The CAA values of β-carotene-loaded multilayer nanoemulsions increased in the following order: PRI-NE (48.36%), TER-SA (72.18%), SEC-CS (77.29%), and TER-DS (87.24%). The CAA values of the TER-SA, SEC-CS, and TER-DS were 1.49-fold, 1.60-fold, and 1.80-fold higher, respectively, than those of the PRI-NE. The results of the CAA assay were similar to the results of the DPPH assay and were more effective in reflecting the differences between the four types of nanoemulsions. The CAA values of the PRI-NE, SEC-CS, and TER-SA were lower than that of the TER-DS, which may be attributed to the excellent water solubility of DS and the sulfate group being combined with the hydrogen atoms of the mucosa, resulting in more ideal solubility and mucoadhesion of the TER-DS.

These results suggest that multilayer nanoemulsions are ideal drug delivery systems that promote cellular antioxidant activity. Moreover, TER-DS was found to be an optimal drug delivery system.

## 4. Conclusions

β-Carotene-loaded oil-in-water multilayer nanoemulsions were created using the layer-by-layer technique to enhance the stability of both emulsions and β-carotene, and to promote the antioxidant activity of β-carotene. Tween 20 was used to stabilize the primary emulsions, onto which CS, DS, or SA were deposited sequentially. The β-carotene-loaded multilayer nanoemulsions had average diameters ranging from 92 to 110 nm and exhibited high EE in the range of 91.9–99.3%. β-Carotene-loaded multilayer nanoemulsions showed significantly higher antioxidant activity and in vitro bioaccessibility than a PRI-NE. With an increase in the number of layers coating β-carotene, the stability, antioxidant ability, and in vitro bioaccessibility were gradually improved. Furthermore, the rate of FFA release from the PRI-NE was slightly higher than that from the SEC-CS, TER-DS, and TER-SA. In particular, the TER-DS exhibited the highest DPPH radical scavenging activity (66.94%), CAA (87.24%), and in vitro bioaccessibility (10.88%), and was composed of 0.25% (*w*/*w*) CS and 1.0% (*w*/*w*) DS. The DPPH, CAA, and bioaccessibility were 1.28-fold, 1.80-fold, and 1.55-fold higher, respectively, than those of the PRI-NE. The abovementioned findings indicate that the stability, antioxidant activity, and in vitro bioaccessibility of β-carotene may be enhanced by multilayer nanoemulsification, with the TER-DS showing optimal properties. Therefore, β-carotene-loaded oil-in-water multilayer nanoemulsions produced using Tween 20, CS, and DS may be effective delivery systems to improve the antioxidant activity, stability, and in vitro digestion of β-carotene.

## Figures and Tables

**Table 1:** Physical properties of optimum PRI-NE, SEC-CS, TER-DS, and TER-SA nanoemulsions.

Nanoemulsions	Particle Size (nm)	Polydispersity Index Value	Zeta Potential (mV)
PRI-NE	92.2 ± 0.7 ^b^	0.163 ± 0.013 ^c^	−42.4 ± 1.7 ^b^
SEC-CS	109.5 ± 2.6 ^a^	0.189 ± 0.020 ^a^	37.3 ± 1.4 ^a^
TER-DS	91.9 ± 1.1 ^b^	0.159 ± 0.016 ^c^	−46.6 ± 1.9 ^c^
TER-SA	92.6 ± 1.0 ^b^	0.176 ± 0.009 ^b^	−53.1 ± 4.8 ^d^

^a–d^ Means with different letters are significantly different (*p* < 0.05). Key: PRI-NE, primary nanoemulsion; SEC-CS, secondary nanoemulsion coated with chitosan; TER-DS, tertiary nanoemulsion coated with dextran sulfate sodium salt; TER-SA, tertiary nanoemulsion coated with alginic acid sodium salt. Measurement was performed in triplicate, and the results are expressed as the mean and standard deviation.

## Data Availability

The original contributions presented in the study are included in the article. Further inquiries can be directed to the corresponding author.
